# Characteristics of women who do not consult a doctor: a population-based study

**DOI:** 10.11606/S1518-8787.2018052000190

**Published:** 2018-05-03

**Authors:** Juvenal Soares Dias-da-Costa, Annie Pozeczek Koltermann, Bruna Cappellesso, Josiele Flores Lisowski, Maiton Bernardelli, Paula Brustolin Xavier, Talita Donatti, Tissiani Morimoto, Fernanda Souza de Bairros, Maria Teresa Anselmo Olinto

**Affiliations:** I Universidade do Vale do Rio dos Sinos. Programa de Pós-Graduação em Saúde Coletiva. São Leopoldo, RS, Brasil; II Universidade Federal de Pelotas. Faculdade de Medicina. Pelotas, RS, Brasil; III Universidade Feevale. Novo Hamburgo, RS, Brasil; IV Universidade do Oeste de Santa Catarina. Área das Ciências da Vida. Joaçaba, SC, Brasil; V Centro Universitário Ritter dos Reis. Curso de Fisioterapia. Porto Alegre, RS, Brasil

**Keywords:** Women, Patient Acceptance of Health Care, Health Services, utilization, Primary Health Care, Health Services Accessibility, Ambulatory Care, Mulheres, Aceitação pelo Paciente de Cuidados de Saúde, Serviços de Saúde, utilização, Atenção Primária à Saúde, Acesso aos Serviços de Saúde, Assistência Ambulatorial

## Abstract

**OBJECTIVE:**

To analyze the prevalence of not consulting a doctor within a year.

**METHODS:**

Cross-sectional population-based study, including women aged 20–60 years, living in the urban area of São Leopoldo, state of Rio Grande do Sul, in 2015. The association between variables and outcome was assessed using prevalence ratios and 95% confidence intervals (95%CI). The adjusted analysis was performed using Poisson regression with robust variance.

**RESULTS:**

Among the 1,127 women participating in the study, 954 (84.6%, 95%CI 82.5–86.7) reported having consulted a physician in the year prior to the interview, 173 (15.4%, 95%CI 13.2–17.5) did not. Women belonging to lower income classes D and E, younger, and smokers had higher prevalences of no medical visits. The participants with hypertension had a higher prevalence of consultations.

**CONCLUSIONS:**

There was no expected evolution in the local health system, despite the emergence of the policies implemented in this period. It is necessary to provide care for those in less favored socioeconomic conditions and for younger women.

## RESUMO

**OBJETIVO:**

Analisar a prevalência de não consultar com médico no período de um ano.

**MÉTODOS:**

Estudo transversal de base populacional, incluindo mulheres de 20 a 60 anos, residentes na zona urbana de São Leopoldo, RS, em 2015. A associação entre as variáveis e o desfecho foi avaliada por meio das razões de prevalência e dos intervalos de 95% de confiança (IC95%). A análise ajustada foi realizada por meio da regressão de Poisson com variância robusta.

**RESULTADOS:**

Entre as 1.127 mulheres participantes do estudo, 954 (84,6%; IC95% 82,5–86,7) referiram consultar com médico no ano anterior à entrevista, 173 (15,4%; IC95% 13,2–17,5) não consultaram. As mulheres inseridas nas classes econômicas D e E, com menor idade, e fumantes apresentaram maiores prevalências de não consulta médica. As participantes com hipertensão arterial tiveram maior prevalência de consultas.

**CONCLUSÕES:**

Não houve a esperada evolução no sistema local de saúde, apesar do surgimento das políticas implantadas nesse período. É necessário provimento da atenção para os indivíduos em condições socioeconômicas menos favorecidas e para as mulheres mais jovens.

## INTRODUCTION

Universal and free access to health services and actions in the Brazilian health model was guaranteed by the Organic Health Law^a^. The implementation of the Unified Health System (SUS) occurred in a decentralization movement, providing greater autonomy for the municipalities, and involving social participation^1^. For the expansion of basic care, which aimed at universal access and referral to more complex levels of care, the Ministry of Health formulated a series of programs. Among them, there is the Family Health Strategy (ESF), which provided greater population coverage^1^ ^,^ ^2^.

Studies that evaluated access to health services evidenced advances after the Sanitary Reform movement and SUS^3^ ^–^ ^5^ implementation. Between 1981 and 2008, the number of individuals using primary care increased by approximately 450%^1^. When comparing data from the National Household Sample Survey (PNAD) between 1998 and 2008, it was found that the probability of having at least one medical consultation in the last 12 months became more equitable over time, and the factors associated with greater equity included health needs, schooling, and bonding with ESF^6^. Currently, Brazil has a 64.3% ESF coverage ratio, with an estimated population coverage of 124,773,082 individuals and 266,583 community health agents working in the health system^b^. Specifically, regarding the use of health services, a study that used the 1998 PNAD showed that women consulted doctors more than men. It was also observed that women over 50, retired or housewives, and with higher income, consulted more often^3^. Another study with PNAD data from 2003 and 2008 found that women consulted doctors more than men. It also showed that women over 50, with higher income and higher self-reported morbidity, also consulted doctors more often^5^.

Two cross-sectional population-based studies were developed, verifying the use of health services in São Leopoldo, state of Rio Grande do Sul. In 2003, a study that enrolled 1,022 women aged 20 to 60 years showed that those enrolled in economic classes C, D, and E, with lower schooling and lower income, showed less use of health services, evidencing iniquities among the most vulnerable population^7^. In turn, another study carried out in 2007 with 1,098 individuals of both genders, aged 20–69 years old, showed that the participants in the intermediate categories of income, schooling, and economic class sought fewer health services, suggesting advances in the local health system in relation to the neediest populations^8^.

In this way, understanding the demand and the use of health services based on epidemiological studies allows us to understand the local reality, identifying vulnerable populations and instrumentalizing the management to plan and provide adequate care.

Therefore, this study aimed to analyze the prevalence of not consulting with a doctor within a year in São Leopoldo, state of Rio Grande do Sul.

## METHODS

A population-based cross-sectional study with a representative sample of women between the ages of 20 and 69 years old residing in the urban area of São Leopoldo, state of Rio Grande do Sul. This article was a cross-section of a research project entitled “Living conditions and health of adult women: a population-based study in the Vale do Rio dos Sinos - Evaluation after 10 years”, conducted by the University of Vale do Rio dos Sinos between February and October 2015. This study was a replication of a similar study conducted in 2003 so that its results could be compared. The sample size calculation was performed in the EpiInfo 6.0 program. They were estimated from the female population of São Leopoldo, with innumerable outcomes including life habits, nutritional and psychological aspects, preventive procedures, contraceptive methods, morbidities and the use of health services. We chose the result that required a larger sample size. The outcome that required a larger sample size was a prevalence of delayed cytopathological examination (5.7%)^9^, with a sampling error of 3%, adding 25% for loss and control of confounding factors, totaling 1,281 women. The sample size for non-use of outpatient health services was estimated from the prevalence of the outcome in non-exposed (6.8%)^7^, with 1:2 ratio of non-exposed to exposed, 2.0 risk ratio, 95% confidence level and 80% power, resulting in 945 individuals.

The sample was systematic. The 371 census tracts in the urban area of São Leopoldo were classified in descending order from the sector with the highest monthly nominal income of persons aged 10 years and older (with or without income), according to the Brazilian Institute of Geography and Statistics (IBGE)^c^. Forty-five census tracts were selected. In each conglomerate, the block and the corner were drawn to start the survey. At each house visited, two residences were jumped to include 36 houses in each tract. All women who met the inclusion criteria were interviewed. Standardized, pre-coded and pre-tested questionnaires similar to the previous study version were applied, with open and closed questions to the participants. Measurements of weight and height of the participants were collected through scales and portable anthropometers.

Women who did not live in the drawn house, pregnant women and those with mental disabilities were excluded from the study.

The interviewers were submitted to the training program to standardize the application of the instruments. Two more attempts were made at different days and times in the case of closed households with absent residents.

Quality control was performed in a random sample of 10% of the people included in the study, to evaluate the internal validity of the research.

The outcome was “did not have any medical appointments in the year prior to the interview” and was evaluated by the question “How many times have you consulted your doctor since <month> last year?”.

The independent variables were classified as demographic, socioeconomic, lifestyle and some morbidities.

Demographic variables were: age (categorized every 10 years), self-reported skin color (white and non-white), and marital status (married/in a union or living alone, single, separated/divorced, widowed).

As socioeconomic variables we used: schooling (≥ 15 years, 11 to 14 years, 8 to 10 years, 5 to 7 years, ≤ 4 years); per capita family income in minimum wages (> 3 minimum wages, 1 to 3 minimum wages, < 1 minimum wage); and economic classification (A; B; C; D+E) according to the economic classification criterion proposed by the Brazilian Association of Research Companies (ABEP)^d^.

The variables that represented the habits of life were: smoking (non-smokers, ex-smokers, smokers); excessive alcohol consumption (no; yes); and physical activity (yes, no). Alcohol consumption was established from the frequency, type of drink and amount consumed, classified as excessive when equal to or greater than 30 g ethanol/day. Participants were considered physically active when they reached at least 150 minutes of physical activity verified through the IPAQ^e^ – short version.

Morbidities were included in the analysis as being overweight (no; yes); diabetes diagnosed by a physician referred by the patient (no; yes); reported systemic arterial hypertension (no; yes); and minor psychiatric disorders (no; yes). The nutritional status of the patients was verified using the body mass index (BMI). It was considered overweight when the BMI reached a value equal to or greater than 25 kg/m^2^. The presence of minor psychiatric disorders was verified through the application of the Self Reporting Questionnaire (SRQ-20). This instrument consisted of 20 dichotomous questions. Participants with a score of seven or more were considered as having minor psychiatric disorders.

Data entry was done in duplicate to avoid typing errors. Characterization of the sample was performed.

The gross analysis indicated the prevalence ratios, with respective 95% confidence intervals and statistical tests. The adjusted analysis was performed by Poisson regression with robust variance, following a hierarchical model. The variables that obtained p = 0.20 in the gross analysis were selected for the adjusted analysis, remaining in the model those with a statistical significance of p < 0.05. The hierarchical model of analysis was composed of demographic variables (age, skin color, marital status) and socioeconomic variables (schooling, economic class, per capita income in minimum wages) in the first level, which determined the variables that represented life habits (smoking, physical activity, and excessive alcohol consumption). At the third level, determined by the variables arranged at the higher levels, there were some morbidities (overweight, diabetes mellitus, hypertension, and minor psychiatric disorders). All variables determined the outcome. We did not include the schooling variable in the analysis adjusted for the possible collinearity since it was contained in the economic class and 51.8% of the interviewees affirmed to be the head of the family.

The statistical analysis was obtained in the SPSS and Stata 12 programs. The design effect was estimated in the Stata program, with a value of 0.95 and was not considered in the analysis. The Project was submitted and approved by the Ethics and Research Committee of the Universidade do Vale do Rio dos (Sinos Protocol 653.394, on May 20, 2014). All participants signed a free and informed consent form.

## RESULTS

Among the 1,127 women participating in the study, 84.6% (95%CI 82.5–86.7) reported having consulted a physician in the year prior to the interview, 15.4 (95%CI 13.2–17.5) reported the opposite. The percentage of losses and refusals reached 11.9%.

There was a homogeneous distribution among the age categories, with a predominance of the age group of 40 to 49 years old (24.5%). The sample consisted mostly of white women (74.4%), married or in a stable union (63.9%), with eight years or more of study (59.4%), belonging to economy class C (53.1%), and with *per capita* family income below one minimum wage (60.8%) (Table 1).

**Table 1:** Demographic and socioeconomic characteristics and gross analysis of the prevalence of no medical consultation among women. São Leopoldo, state of Rio Grande do Sul, Brazil, 2015.

Variable		n	%	Prevalence of non-use (%)	PR	95%CI	p
Age (years)							< 0.001
	20–29	216	19.2	51 (29.3)	1		
	30–39	244	21.7	44 (25.3)	0.76	0.53–1.09	
	40–49	276	24.5	41 (23.6)	0.63	0.43–0.91	
	50–59	228	20.2	23 (13.2)	0.43	0.27–0.67	
	60–69	163	14.5	15 (8.6)	0.39	0.23–0.67	
Color							0.78
	Non-white	288	25.6	46 (16.0)	1		
	White	839	74.4	128 (15.3)	0.95	0.69–1.29	
Marital status							0.44
	Single	227	20.1	43 (18.9)	1		
	Married/In a union	720	63.9	105 (14.6)	0.79	0.57–1.09	
	Separated/Divorced	110	9.8	16 (14.5)	0.78	0.46–1.33	
	Widow	70	6.2	10 (14.3)	0.76	0.40–1.43	
Education (years)							< 0.01
	≥ 15	110	9.8	6 (5.5)	1		
	11–14	360	32.0	62 (17.2)	3.16	1.40–7.10	
	8–10	198	17.6	40 (20.2)	3.61	1.58–8.26	
	5–7	253	22.5	42 (16.6)	3.04	1.33–6.95	
	0–4	204	18.1	24 (11.8)	2.16	0.91–5.12	
Economic class							
	A	44	3.9	2 (4.5)	1		< 0.001
	B	346	30.9	37 (10.7)	2.35	0.59–9.43	
	C	595	53.1	101 (17.1)	3.73	0.95–14.64	
	D+E	136	12.1	33 (24.3)	5.34	1.34–21.36	
Income *per capita* in minimum wages (MW)							< 0.001
	3 or more	66	6.1	7 (10.6)	1		
	1 a 2.99	361	33.1	32 (8.9)	0.83	0.38–1.81	
	Less than 1	663	60.8	127 (19.3)	1.81	0.88–3.71	

PR: prevalence ratio

The highest prevalences of no medical consultation occurred in the younger women in the gross analysis. Women with less than 14 years of schooling presented a higher prevalence of no consultation, but this effect was not observed in the category of up to four years. The economic classes D and E presented higher prevalences of not consulting when compared to women enrolled in class A. Women with a *per capita* family income lower than one minimum wage had a higher prevalence of no consultations. However, the confidence intervals included the unit value, although the test result was significant, referring the variable to the adjusted model. There were no statistically significant differences in the variables skin color and marital status (Table 1).

Most women did not smoke (59.0%), did not consume excessive alcohol (97.0%), and did not practice physical activity (85.6%). A total of 66.1% of the participants were positive for overweight, 28.1% for hypertension, 8.1% for diabetes mellitus, and 39.8% for minor psychiatric disorders (Table 2).

**Table 2:** Characteristics of life habits and morbidities and gross analysis of the prevalence of no medical consultation among women. São Leopoldo, state of Rio Grande do Sul, Brazil, 2015.

Variable		n	%	Prevalence of non-use (%)	PR	95%CI	p
Smoking							0.001
	Does not smoke	661	59.0	90 (13.6)	1		
	Former smoker	253	22.6	32 (12.6)	0.93	0.64–1.35	
	Smoker	207	18.5	207 (24.6)	1.81	1.33–2.46	
Excessive consumption of alcohol							0.17
	No	1,086	97.0	166 (15.3)	1		
	Yes	34	3.0	8 (23.5)	1.54	0.83–2.87	
Physical activity							0.25
	Yes	162	14.4	20 (12.3)	1		
	No	965	85.6	154 (16.0)	1.29	0.84–2.00	
Overweight							0.001
	No	380	33.9	78 (20.5)	1		
	Yes	741	66.1	95 (12.8)	0.62	0.47–0.82	
High blood pressure							< 0.001
	No	810	71.9	153 (18.9)	1		
	Yes	316	28.1	21 (6.6)	0.35	0.16–0.22	
Diabetes Mellitus							< 0.01
	No	1,029	91.9	167 (16.2)	1		
	Yes	91	8.1	5 (5.5)	0.34	0.14–0.19	
Psychiatric disorders							0.65
	No	678	60.2	102 (15.0)	1		
	Yes	449	39.8	72 (16.0)	1.06	0.81–1.40	

PR: prevalence ratio

Female smokers had a higher prevalence of the outcome in the gross analysis. Women classified as having excessive alcohol consumption apparently had a higher prevalence of no consultation, but no difference in the statistical test. In turn, overweight, hypertensive, and diabetes mellitus women were protected from no consultation. Minor psychiatric disorders were not associated with the outcome (Table 2).

In the adjusted analysis of the first level, age, economic class, and per capita income remained associated with the no medical consultation outcome. From the age of 50, there was 58.0% protection for no consultations compared to women aged 20 to 29 years. The prevalence of no consultation among the women enrolled in classes D and E was 7.2 times higher than those in economy class A. Despite the imprecision of the confidence intervals of income categories, the variable was kept in the model due to the value of the test. Even adjusted for the variables of the first level, female smokers showed a higher prevalence of the outcome. After adjusting for age, economic class, family income, smoking and among variables of the same level, women with hypertension remained protected from no consultation (Table 3).

**Table 3:** Adjusted analysis of the prevalence of no medical consultation among women. São Leopoldo, state of Rio Grande do Sul, Brazil, 2015.

Variable		Adjusted PR	95%CI	p
Age group (years)^a^				< 0.001
	20–29	1		
	30–39	0.76	0.53–1.09	
	40–49	0.68	0.47–0.98	
	50–59	0.42	0.26–0.68	
	60–69	0.42	0.25–0.72	
Economic class^a^				< 0.05
	A	1		
	B	4.2	0.61–28.5	
	C	5.76	0.84–39.5	
	D+E	7.20	1.03–50.3	
Income *per capita* in minimum wages (MW)^a^				0.02
	3 or more	1		
	1 a 2.99	0.59	0.28–1.25	
	Less than 1	1.00	0.47–2.11	
Smoking^b^				0.02
	Does not smoke	1		
	Former smoker	0.95	0.64–1.41	
	Smoker	1.53	1.10–2.13	
Excessive consumption of alcohol^b^				0.79
	No	1		
	Yes	1.10	0.55–2.20	
Overweight^c^				0.06
	No	1		
	Yes	0.76	0.57–1.01	
High blood pressure^c^				< 0.001
	No	1		
	Yes	0.40	0.24–0.66	
Diabetes mellitus^c^				0.25
	No	1		
	Yes	0.63	0.25–1.57	

Adjusted RP: adjusted prevalence ratio

a Variables adjusted to each other.

b Adjusted for age group, economic class, *per capita* income in minimum wages, and to each other.

c Adjusted for age group, economic class, *per capita* income in minimum salaries, smoking, and to each other.

## DISCUSSION

Several policies seek to improve the socioeconomic conditions of the Brazilian population in recent years^10^. The Gini index, which reflects the level of social inequality, has decreased significantly and the implementation of the SUS is considered as a catalyst for improving the health conditions of the Brazilian population^11^. From the point of view of health policies, we must highlight the attempt to strengthen primary health care through the ESF. It certainly was a way of increasing access and guaranteeing universality of care, showing impressive expansion and changes in health indicators^12^. Thus, it was expected to find positive results when replicating a cross-sectional population-based study on the use of health services in São Leopoldo over the 10-year period.

It was possible to compare the results of the socioeconomic variables of the present research with another study carried out in 2003^7^, emphasizing that the previous research was restricted to the population from 20 to 60 years old.

Changes were observed in comparison to the socioeconomic variables of the two studies. In 2003, 66.9% of the participants were classified as economic class C (39.3%) and D+E (27.6%). In the present study, 53.1% of the women were enrolled in class C and 12.1% in the D+E category, corresponding to an increase of 35.0% and a decrease of 56.1%, respectively. In 2003, women in the D+E category had a higher prevalence of no consultation (16.7%). In this study, the prevalence of no consultation reached 24.3%, indicating an increase of 45.5%. This represented an increase in people in class C, but the effect of no consultation on the women from the lowest economic class continued.

In 2003, 78.9% of the participants reported receiving up to three minimum wages, while in the present study, this contingent reached 93.9%. This corresponds to an increase of 19.0%. In addition, the category of up to one minimum wage covering 36.7% of the women in the first study reached the 60.8% percentage in the present investigation, representing an increase of 65.6%. In 2003, women in the lowest income category had a higher prevalence of no consultation. However, the confidence intervals did not confirm the difference from the outcome in the present study.

In 2003, 20.3% of women reported up to four years of study. In the present study, this percentage decreased to 18.1%, with a decrease of 10.0%.

The changes in the distribution of the variable economy class and schooling with a decrease of the percentages in the worst categories can be understood as a consequence of social policies implanted in the country in the period. Thus, the growth of participants in class C can be explained by the possession of consumer goods and schooling. However, even after 10 years, the health system did not overcome the inequities found in 2003.

In the present study, 15.4% (95%CI 13.2–17.5) of the women did not consult a doctor in the last 12 months. The prevalence of non-use of outpatient health services in the last 12 months was 13.3% (95%CI 11.2–15.4) in a study with the same design, conducted in 2003, with a similar population. However, the previous study considered any use of an outpatient health service, unlike the current research, where participants were specifically asked about medical consultation. Even so, there was no expected evolution in the local health system, despite the emergence of policies implemented throughout this period (2003 to 2015).

Other studies were carried out in the southern region of Brazil with similar results to the present study. In Santa Catarina, a prevalence of 76.0% (95%CI 73.6–78.4) was observed in the adult population. Thus, it was inferred that the prevalence of no medical consultation in the population was 24.0% and 18.3% among women^13^. In the study by Bastos et al., conducted in a low-income community in Porto Alegre, state of Rio Grande do Sul, the prevalence of medical consultation was 76.2% (95%CI 74.8–77.6) in the last year and 64.8% (95%CI 63.0–66.7) in the last three months, higher among women (67.8%) than among men (60.2%)^14^.

Studies that analyzed the great national health surveys carried out in the country also presented similar results. Macinko and Lima-Costa, evaluating horizontal equity trends in the use of health services in the years 1998, 2003 and 2008 with PNAD data, showed that the probability of having at least one medical consultation in the last 12 months became more equitable over time^6^. However, the prevalence of medical consultation was 54.5% in 1998, 63.8% in 2003, and 67.7% in 2008, all below the percentage reached in the present study^6^. One of the findings of the study was that the greater inequality in the use of the service is related to income, indicating inequity of the health system. More recent data, using the National Health Survey of 2013, pointed out that the prevalence of not consulting a doctor during the last year was 25.8% among the interviewees and 18.0% among women^15^.

In the present study, the prevalence of no consultation among women in the D and E classes was seven times higher than those in class A. In the cross-sectional study previously carried out in São Leopoldo, it was identified that women in economic class D and E used health services 2.4 times less when compared to those in class A^7^. The results of both studies suggested that the most vulnerable women had greater difficulty in accessing health services and that this association was even more evident when seeking medical care.

These findings are consistent with national comparisons, all of which evidence the persistence of health inequities. In a cross-sectional study carried out in Porto Alegre, women belonging to economic classes C, D, and E consulted doctors less^14^. The under-utilization outcome applied by Boccolini and Souza Júnior^15^ was the occurrence of a positive response in at least one combination of the following questions: never consulted a physician; never consulted a dentist, and never measured blood pressure or blood glucose^15^. The results of the adjusted analysis showed greater underutilization in social classes C, D and E, and in individuals with lower schooling^15^. The present study confirmed the persistence of undesirable iniquity in the health system of São Leopoldo.

Women aged 20 to 29 years old had a lower probability of not having a medical consultation, which confirms the findings of other studies^8^ ^,^ ^14^ ^,^ ^16^. Chronic noncommunicable diseases are more prevalent in elderly individuals,^16^ ^,^ ^17^ which requires greater intensity of medical care. In addition, secondary prevention programs targeted at women's health primarily target older age groups.

Among the findings of the present study, we found that participants who smoked had a higher prevalence of no medical consultations. In a study conducted in Lages, state of Santa Catarina, Boing et al.^13^ also showed that smokers consulted doctors less.

Studies indicate that the presence of chronic non-communicable diseases or that the presence of more morbidities implies increased health care and consequently more consultations^3^ ^,^ ^4^. Among the morbidities analyzed in the present study, differences were found only among women who had hypertension. In the 2003 study, the same morbidities were not associated with outcome^7^. However, other studies show a greater use of health services in patients with diabetes mellitus^13^ ^,^ ^18^, minor psychiatric disorders^4^ ^,^ ^18^, and arterial hypertension^19^. Participants with these morbidities would be expected to report more consultations. Thus, the findings of the present study point to the need to implement programmatic actions to capture users with these diseases.

These findings may be related to poor health coverage. The proportion of population coverage by the ESF in São Leopoldo was 20.7% in October 2016. According to criteria for the classification of population coverage by the ESF, values below 30.0% coverage can be classified as incipient^19^.

The present study was conducted with the necessary rigor for population-based investigations. We identified changes in the socioeconomic structure of the municipality, noting iniquities in the use of medical consultations. This study highlighted the characteristics of women who did not consult physicians, drawing attention to the need to point out the care directed to chronic non-communicable diseases. Thus, the present study emphasizes the convenience of the provision of care for individuals with less favored socioeconomic status and in younger women.

## Footnotes

a Brasil. Lei n° 8.080, de 19 de setembro de 1990. Lei Orgânica da Saúde. Dispõe sobre as condições para a promoção, proteção e recuperação da saúde, a organização e o funcionamento dos serviços correspondentes e dá outras providências. *Diario Oficial Uniao*. 20 set. 1990; Seção 1:18055.

b Ministério da Saúde (BR), Secretaria de Atenção à Saúde, Departamento de Atenção Básica, Coordenação Geral de Gestão da Atenção Básica. Histórico de cobertura de Saúde da Família. Brasília (DF); 2016 [cited 2016 Dec 10]. Disponível em: http://dab.saude.gov.br/dab/historico_cobertura_sf/historico_cobertura_sf_relatorio.php

c Fundação Instituto Brasileiro de Geografia e Estatística. Censo Demográfico 2010. Rio de Janeiro: IBGE; 2010 [cited 2016 Dec 12]. Disponível em: https://censo2010.ibge.gov.br/

d Associação Brasileira de Empresas de Pesquisa. Critério de Classificação Econômica Brasil. São Paulo: ABEP; 2016 [cited 2016 Dec 12]. Disponível em: http://www.abep.org/codigosguias/ABEP_CCEB.pdf

e The IPAQ Group. International Physical Activity Questionnaire – IPAQ [citado 10 dez 2016]. Disponível em: https://sites.google.com/site/theipaq/
